# Eclectic Department

**Published:** 1851-11

**Authors:** 


MOUNT JOY, Lan. Co., Pa., Sept. 4th, 1851.

*Mr. Editor*—There is in a late number of your Gazette, an article assuming the views of Dr. Reid, of Rochester, N. Y., on dislocation of the femur. These views are, as asserted, novel and important, and I add, absolutely true, founding my assurance on the actual example of an experiment introduced to my notice, by Dr. D. Bly, at the time a fellow-student in the Jeff. Med. Coll. Sess. of 1850 and '51, and whose graduating essay was founded on the subject in question. If I remember aright, this gentleman was a student of Dr. Reid; and, in his laudable ambition to produce an excellent thesis on this almost newly-known subject, he experimented with untiring zeal, during the last summer of his preparatory studies, to discover what amount of extension beyond the normal length, the muscles engaged in dislocations would bear; and collateral evidences, the results of the experiments on the above, are expressed in his thesis, and from these are deduced the unavoidable conclusions to follow. To be certain that there was no possibility of mistake in asserting what has been purported in the conclusions of Dr. Reid's proposition, Dr. Bly, by several dissections, evidenced to his own and the minds of the few who witnessed his experiments, that the *rule* could be depended upon. He dislocated the head of the femur, and by abducting and rotating movements, as described in Dr. Reid's 4th proposition, the ball rolled around the lip of the acetabulum and slipped into the cavity when the last point of abduction was made. These experiments demonstrated conclusively the feasibility of the plan; for, allowing a large amount of force to be overcome in a living case, where the energies are superior to that experienced in working on the tissues deprived of vitality, as the experiment was necessarily tried with, yet the force of the surgeon is able to overcome, with ease, the dislocation. My friend, Dr. B., informed me, he had witnessed the operation as performed by Dr. Reid in the summer of 1850, and assured me there was little more force exerted in the living than we found in the dead example. The muscles engaged in contraction at the commencement of rotation, are overcome with the enormous force exerted by the lever, the femur becomes, and after passing a certain angle, (easily recognized in the progress of the replacing movements,) the muscles, before contracting and drawing the head of the femur against the lips of the acetabulum at a disadvantage, become auxiliaries to the reduction, overcoming the power of the small muscles, which though acting in a natural direction, yet do not exert their opposite and resisting powers forcibly, being scarcely elongated at all; the flection of the thigh keeping the point of their insertion at an almost regular distance, whilst the ball revolves around the circumference of the acetabulum: the muscles which arise from the pelvic origins are put on the stretch at the latter period of the abducting movement, but they present no difficulty to adjustment when the relaxing direction to the superior muscles is remembered and given or dispensed with. A gliding motion, and perhaps snapping slip, assures to the surgeon the conclusion of the much dreaded operation. My friend informed me, he had discovered the author of the views he sustained, to be an old physician in one of the eastern states; that his preceptor, Dr. Reid, revived the idea, and elicited what is justly due him, applause, and credence for successfully applying so simple means to so hitherto formidable an operation. But I cannot leave this notice and tribute to my fellow-student, without expressing regrets that he should have deposited in the archives of his alma mater, than whom there is none worthier, his labored and instructive thesis, which most certainly would do credit to its author, and exhibit to the surgeon a talisman against these terrible luxations. The foregoing will be recognized by the friends of the two gentlemen interested, as not derogatory to the commmunication and claims of Dr. R., nor claiming too much of the guerdon-honor to Dr. B., whose want of ardor has kept him from arrogating to himself his just due. As regards the extension of such practice as avowed, my examinations lead me to believe the operation will ever be found successful, especially in recent cases; and, until greater objections appear than has hitherto been raised, we (the friends of the operation) will remain sic sentis, sic sentiam.

— *N. Y. Med. Gazette.* VERITAS.

